# Isolated Retroperitoneal Hydatid Cyst Invading Splenic Hilum

**DOI:** 10.1155/2014/303401

**Published:** 2014-03-26

**Authors:** Safak Ozturk, Mutlu Unver, Burcin Kibar Ozturk, Eyup Kebapci, Osman Bozbiyik, Varlık Erol, Nihat Zalluhoglu, Mustafa Olmez

**Affiliations:** ^1^Department of General Surgery Clinic, T.C.S.B. Tepecik Teaching and Research Hospital, 35110 Izmir, Turkey; ^2^Department of Radiology, Faculty of Medicine, Ege University, Izmir, Turkey

## Abstract

*Introduction*. Hydatid disease (HD) is an infestation that is caused by the larval stage of *Echinococcus granulosus*. The liver is affected in approximately two-thirds of patients, the lungs in 25%, and other organs in a small proportion. Primary retroperitoneal hydatid cyst is extremely rare. The most common complaint is abdominal pain; however, the clinical features of HD may be generally dependent on the location of the cyst. *Case Presentation*. A 43-year-old female was admitted with the complaint of abdominal pain. Her physical examination was normal. Computed tomography (CT) revealed a 17 × 11 cm cystic lesion, with a thick and smooth wall that is located among the left liver lobe, diaphragm, spleen, tail of the pancreas, and transverse colon and invading the splenic hilum. Total cystectomy and splenectomy were performed. Pathological examination was reported as cyst hydatid. *Discussion*. Cysts in the peritoneal cavity are mainly the result of the spontaneous or traumatic rupture of concomitant hepatic cysts or surgical inoculation of a hepatic cyst. Serological tests contribute to diagnosis. In symptomatic and large hydatid peritoneal cysts, surgical resection is the only curative treatment. Total cystectomy is the gold standard. Albendazole or praziquantel is indicated for inoperable and disseminated cases. Percutaneous aspiration, injection, and reaspiration (PAIR) technique is another nonsurgical option.

## 1. Introduction

Hydatid disease (HD) is an infestation that caused by the larval stage of* Echinococcus granulosus* [1]. It is prevalent in the Middle East, the Mediterranean region, particularly in sheep-raising countries, Australia, Argentina, and Africa. The main hosts are dogs that pass eggs into their feces. Intermediate hosts, for example, sheep, goats, cattle, and human, ingest the eggs and develop cyst formation [2]. Human is the accidental intermediate host in the life cycle of* Echinococcus granulosus* [3]. The annual incidence of HD has been reported as 18 to 20 cases per 100.000 inhabitants [4]. The larval form of* Echinococcus granulosus* enters to the lymphatic circulation via penetrating the intestinal mucosa and it is transported to the liver, lungs, and other organs [1]. The liver is affected in approximately two-thirds of patients, the lungs in approximately 25%, and other organs including brain, muscles, ovaries, spleen, kidneys, bones, heart, and the pancreas in a small proportion [5]. Primary retroperitoneal hydatid cyst is extremely rare and only occasional cases have appeared since Lockhart and Sapinza first described this entity in 1958 [6]. 85% to 90% of patients with* Echinococcus granulosus* infection have single-organ involvement and more than 70% of patients have only one cyst [5]. The cysts may be uni or multiloculated and thin or thick walled. HD is seen more frequently at the ages of 20 to 40 years and usually occurs in childhood and grows so slowly about 1–3 cm per year that the organism may take up to 20 years to reach considerable size [7]. The most common complaint is abdominal pain; however, the clinical features of HD may be nonspecific and generally depends on the location of the cyst [1, 5]. HD in extrahepatic locations especially in the retroperitoneum usually remains asymptomatic unless the cyst grows and produces symptoms due to pressure, rupture of the pleural or peritoneal cavity, secondary infection, or an allergic reaction [2, 6].

## 2. Case Presentation

**Figure 1:**
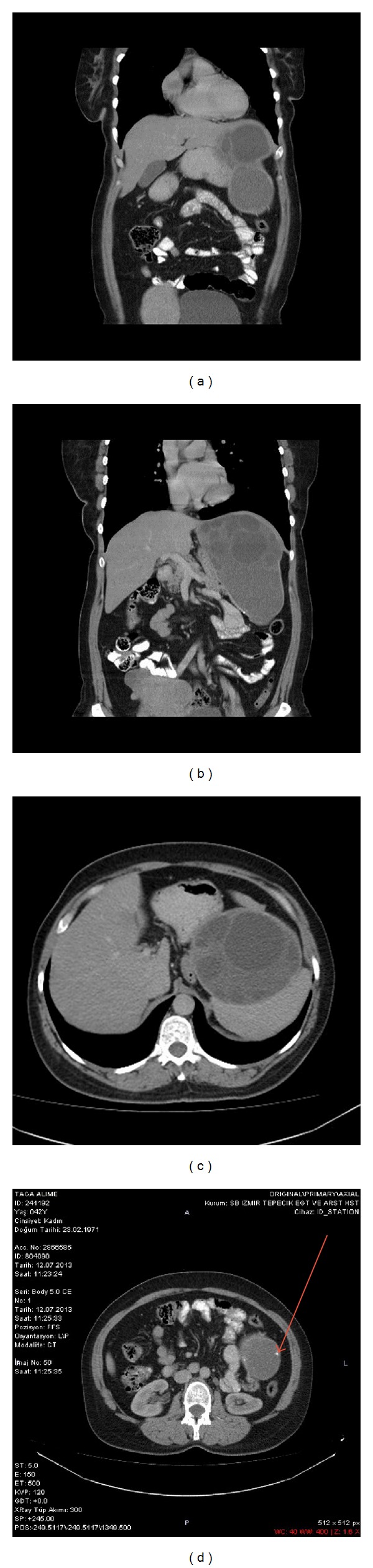
(a) The coronal CT image of bilobar hydatid cyst lesion. (b) The coronal CT image of multiple daughter cysts. (c) The axial CT image of multiple daughter cysts. (d) The axial CT image of inferior portion of the cyst that the red arrow shows the cystic wall calcification.

**Figure 2:**
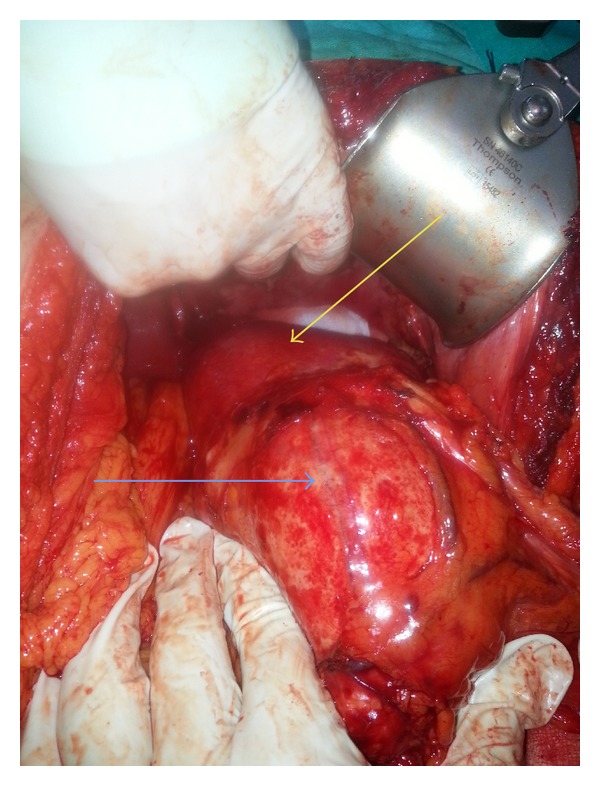
A large cystic mass that is attached to the left liver lobe (yellow arrow shows the left liver lobe and blue arrow shows the cystic mass).

**Figure 3:**
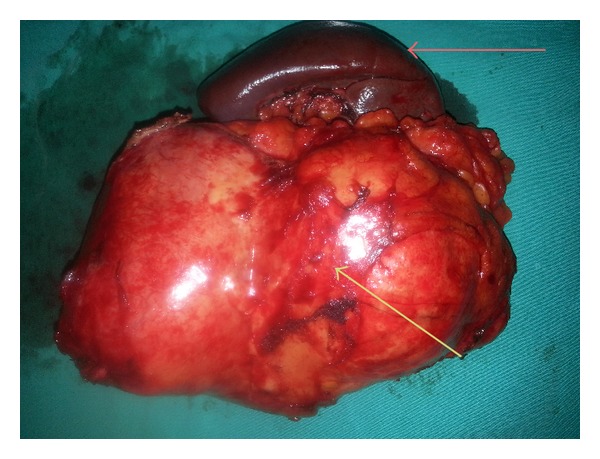
Total cystectomy and splenectomy specimen (red arrow shows the spleen and yellow arrow shows the cystic mass).

A 43-year-old female was admitted to our clinic with the complaint of abdominal pain localized in the left upper quadrant for the last 6 months. Her physical examination was normal and we could not find any palpable masses on abdominal exam. Routine blood analyses revealed a white blood cell count of 7900/mm^3^ and eosinophil count of 30 (35%). Other biochemical investigations were normal. Indirect hemagglutination test (IHAT) for HD was 1/1280 (+). Chest X-ray revealed no pathological signs. An abdominal ultrasonography (USG) showed a 15 × 11 cm cystic lesion that extended from the left liver lobe to the transverse colon. Computed tomography (CT) revealed a 17 × 11 cm cystic lesion, with a thick and smooth wall that is located among the left liver lobe, diaphragm, spleen, tail of the pancreas, and transverse colon and invading the splenic hilum without any pathology of the intraabdominal organs (Figure 1). The patient underwent a laparotomy with a median superior and left subcostal incision. A large cystic mass was identified retroperitoneally, attached to the left liver lobe, diaphragm, mesenterium of the transverse colon, tail of the pancreas, and spleen with a splenic hilum invasion (Figure 2). In order to protect peritoneal soilage, the abdomen was packed with 10% hypertonic saline soaked pads and total cystectomy and splenectomy were performed (Figure 3). Mean operative time was 150 minutes and mean blood loss was 150 cc. The patient was discharged after 7 postoperative days. Pathological examination of the specimen was reported as cyst hydatid. An abdominal CT obtained 3 months following surgery did not reveal any recurrence of cyst.

## 3. Discussion

The retroperitoneal hydatid cyst is rare even in endemic areas [4]. The overall frequency of peritoneal echinococcosis is approximately 13% of all cases [5]. Cysts in the peritoneal cavity are mainly the result of the spontaneous or traumatic rupture of concomitant hepatic cysts or surgical inoculation of a hepatic cyst [1–5, 7]. The spontaneous asymptomatic microruptures of hepatic cysts into the peritoneal cavity are not uncommon [5]. An isolated retroperitoneal hydatid cyst could be caused by haematogenous dissemination of protoscoleces after bypassing the liver and the lungs or by the gastrointestinal tract into the lymphatic system [1, 4]. The differential diagnosis of retroperitoneal cysts also includes soft tissue tumors, retroperitoneal abscess, cystic lymphangioma, embryonal cyst, ovarian neoplasms, teratoma, and other cystic and necrotic solid tumors [1, 4, 6]. Especially in endemic regions such as Turkey, the hydatid cyst must always be considered in the differential diagnosis of cystic lesions [1, 5]. The hydatid cyst is usually asymptomatic and the clinical presentation of HD depends on the organs involved, the size of the cysts, their site within the affected organ, the presence of cyst rupture, spread of protoscoleces, and bacterial infection-related complications [1, 6, 7]. The definitive diagnosis of a retroperitoneal hydatid cyst requires a combined assessment of clinical, radiological, and serological analyses [1, 8]. Routine laboratory tests including complete blood counts and liver function tests are generally normal and nonspecific but eosinophilia occurs in 25% of cases [5–7]. Serological tests contribute to diagnosis. Immunoglobulin G antibody detection by enzyme-linked immunosorbent assay (ELISA) has a sensitivity of 95% and a specificity of 94%. The sensitivity of indirect hemagglutination test (IHAT) has been found to be 87.5% [2, 6]. Radiography, ultrasonography (USG), and computed tomography (CT) studies are important for diagnosis of HD [2, 6]. HD can demonstrate varying imaging features according to the growth stage of the cyst, associated complications, and affected organs [5, 7]. The sensitivity of USG in diagnosing abdominal hydatid cyst ranges from 93% to 98% [1, 2]. CT confirms the diagnosis by revealing the presence of daughter cyst and plaque-like calcifications in the cystic wall and is also superior to USG in detecting the extrahepatic cysts [2, 5, 6]. The sensitivity of CT ranges from 90% to 97% [2]. The management of extrahepatic HD is based on considerations regarding the size, location, and manifestations of the cysts and the overall health status of the patient. Asymptomatic small cysts can be treated with antihelminthic drugs with a usage of 28 days in one to eight repeating cycles, separated with 2-3 weeks of drug-free intervals [2]. In symptomatic and large hydatid peritoneal cysts, surgical resection is the only curative treatment [2, 8]. Surgical treatment can be either radical or conservative. Total cystectomy is the gold standard [2, 6, 7]. For peritoneal cysts which were attached to the intraperitoneal viscera, unroofing and drainage are recommended [2, 7]. The most important thing is to isolate the abdominal cavity with gauzes soaked in 20% hypertonic saline solution for preventing the secondary hydatidosis and allergic reaction [2]. Laparoscopic approaches are also described. Spillage of the cyst contents must be avoided and scolicidal agents must be used in either conventional or laparoscopic technique. Although a variety of scolicidal agents have been used, there is no consensus on which is the best agent. Hydrogen peroxide and 10% povidone-iodine have strong scolicidal activity in experimental models. Albendazole or praziquantel is indicated for inoperable and disseminated cases. Percutaneous aspiration, injection, and reaspiration (PAIR) technique is another nonsurgical option. However, there have been some limitations for PAIR and it is only suitable for predominantly fluid and nonruptured cysts [7].

In conclusion, the possibility of HD in a patient presenting with a retroperitoneal cystic mass should be suspected especially in endemic areas such as sheep-raising Mediterranean Countries and the definitive diagnosis may require surgical removal of the cyst and histopathological examination of the resected specimen. Total cystectomy is the gold standard. When the complete resection is not feasible, unroofing and drainage followed by adjuvant antihelminthic therapy must be performed to prevent secondary recurrence of the cyst.
